# Metabolic Characterization of Deceased Donor Kidneys Undergoing Hypothermic Machine Perfusion Before Transplantation Using ^13^C-enriched Glucose

**DOI:** 10.1097/TXD.0000000000001736

**Published:** 2024-12-10

**Authors:** Kamlesh Patel, Jay Nath, Thomas Smith, Tom Darius, Alpesh Thakker, Sarah Dimeloe, Nicholas Inston, Andrew Ready, Christian Ludwig

**Affiliations:** 1 Department of Renal Surgery, Queen Elizabeth Hospital Birmingham, Birmingham, United Kingdom.; 2 Department of Metabolism and Systems Science, School of Medical Sciences, College of Medicine and Health, University of Birmingham, Birmingham, United Kingdom.; 3 Department of Renal Transplantation, Southmead Hospital, Bristol, United Kingdom.; 4 Surgery and Abdominal Transplant Unit, Department of Surgery, University Clinics Saint Luc, Université Catholique de Louvain, Brussels, Belgium.; 5 Institute of Immunology and Immunotherapy, School of Infection, Inflammation and Immunology, College of Medicine and Health, University of Birmingham, Birmingham, United Kingdom.

## Abstract

**Background.:**

The provision of a metabolic substrate is one mechanism by which hypothermic machine perfusion (HMP) of kidneys provides clinical benefit. This study aimed to describe *de novo* metabolism in ex vivo human kidneys undergoing HMP before transplantation using ^13^C-labeled glucose as a metabolic tracer.

**Methods.:**

Cadaveric human kidneys were perfused with modified clinical-grade perfusion fluid (kidney perfusion solution [KPS-1], Organ Recovery Systems), in which glucose was uniformly enriched with the stable isotope ^13^C ([U-^13^C] glucose). The sampled perfusion fluid was analyzed using a blood gas analyzer, and metabolic profiling was performed using 1-dimensional and 2-dimensional nuclear magnetic resonance spectroscopy and mass spectrometry. Functional outcome measures included serum creatinine levels and the development of delayed graft function.

**Results.:**

Fourteen kidneys were perfused with the modified KPS-1 and successfully transplanted. The mean duration of HMP was 8.7 h. There was a sustained increase in the conversion of glucose into *de novo* glycolytic end products, such as lactate, in donor kidneys during HMP. There was no significant association between functional outcomes and metabolism during the HMP. *De novo* anaerobic metabolism was indicated by continuing lactate production, as indicated by increasing concentrations of universally ^13^C-labeled lactate ([U-^13^C] lactate) in perfusion fluid from all kidneys. This was more evident in donation after circulatory death donor kidneys.

**Conclusions.:**

Our study is the first to use [U-^13^C] glucose to describe the metabolism during HMP. The consequences of an initial warm ischemic insult on circulatory death in donor kidneys continue during the preservation period.

Hypothermic machine perfusion (HMP) describes the recirculation of chilled preservation solution through the renal vasculature of a donor kidney in the hours before renal transplantation. The process has been widely used in clinical practice for over a decade, providing an alternative to static cold storage (SCS). HMP of deceased donor kidneys has become the gold standard of organ preservation in many countries, following the demonstration of improved outcomes after transplantation,^1-7^ when compared with SCS.

The mechanisms through which HMP produces beneficial effects are not fully understood but are likely multifactorial. In addition to suspected endothelial protective effects due to pulsatile flow, flow-induced reductions in intrarenal vascular resistance^8^ are likely to provide benefits through mechanical vasodilatation and molecular vasoprotection.^9-14^ Furthermore, metabolic factors related to the perfusion process may also play a significant role^15^ despite the hypothermic environment, which intuitively may be thought to reduce the impact of ongoing metabolism.

The detection and quantification of active metabolic pathways occurring during HMP can be performed using different types of nuclear magnetic resonance spectroscopy (NMR). Untargeted 1-dimensional proton NMR (1D-^1^H NMR), which defines concentrations of metabolites, has been used in both clinical settings and animal models, demonstrating that metabolite concentrations in circulating allograft HMP perfusate may correlate with subsequent graft function.^15-17^ However, metabolites considered potentially predictive of outcome have inconsistent between studies.^18^ The principal limitation of untargeted studies is that it is a surrogate indicator of parenchymal metabolic pathway activity based on the presence of and change in concentration of metabolites identified in the circulating perfusion fluid.

By contrast, targeted metabolomic studies using 2-dimensional (2D)-^1^H,^13^C heteronuclear single quantum coherence (HSQC) NMR spectroscopy allow direct identification of metabolites developing *de novo* from the parenchymal tissue during HMP via the use of a metabolomic tracer included in the perfusion fluid, a technique known as substrate labeling.

Hence, against this background, in this study, HMP is performed with University of Wisconsin-machine perfusion solution (UW-MPS) enriched with glucose containing the nonradioactive isotope carbon 13 (^13^C) at all 6 molecular loci ([U-^13^C] glucose). During HMP, metabolites derived from ongoing [U-^13^C] glucose metabolism are observed using 2D HSQC NMR spectroscopy.

In one of our previous studies in a porcine HMP model,^19^ kidneys were perfused with UW-MPS containing [U-^13^C] glucose. These studies showed unequivocal evidence of *de novo* metabolism, as demonstrated by ^13^C-labeled glycolytic end products [U-^13^C] lactate and [U-^13^C] alanine. In addition, ^13^C-enriched glutamate was identified at much lower concentrations in the perfusate and tissue samples, indicative of ongoing tricarboxylic acid (TCA) cycle activity and aerobic metabolism.^19^ Such aerobic metabolism was enhanced with the addition of supplemental oxygen during HMP.^20^

The primary aim of this study, also using [U-^13^C] glucose as a metabolic tracer, was to extend these studies for the first time to the human situation by defining *de novo* metabolism occurring during HMP in ex vivo deceased donor kidneys, which then undergo transplantation.

A secondary aim was to identify differences in the metabolic profiles of kidneys from donation after brainstem death (DBD) donor and donation after circulatory death (DCD) donor kidneys, in which differences in donor physiology and ischemic insults the retrieval processed would be considered likely to impact on any ongoing metabolism during HMP. An additional aim was to identify any correlation between metabolic activity and post-transplant clinical outcomes.

## MATERIALS AND METHODS

### Hypothermic Machine Perfusion

#### Recruitment

After kidney recipient recruitment, deceased donor kidneys were perfused under standard HMP conditions at the Queen Elizabeth Hospital, Birmingham, United Kingdom. The implanting surgical team made the decision to initiate HMP after SCS for organ transport had concluded and before recipient recruitment. The duration of the perfusion period was dictated by the timing of the subsequent transplant surgery.

All kidneys were unpaired adult organs subsequently transplanted successfully into adult recipients on the national UK Transplant Waiting List. Pediatric donor kidneys and kidneys comprising part of a multiorgan transplant were excluded from the study.

### Organ Perfusion

Deceased donor kidneys were perfused with 1 L of Kidney Perfusion Solution (KPS-1, Organ Recovery Systems, USA), in which the usual constituent glucose (10 mM) was replaced with [U-^13^C] glucose (10 mM), resulting in a formulation biochemically equivalent to the industry standard UW-MPS. Either version 1.0 or version 1.1 LifePort Kidney Transporters (Organ Recovery Systems (Itasca, IL) were used for HMP, with a constant perfusion pressure of 30 mm Hg, as per standard clinical practice.

### Ethical Approval

Ethical approval for this single-center study was obtained from the National Research Ethics Service Committee (15/EM/0328) and the Hospital Research and Development Department. All patients received standard posttransplant care with immunosuppression according to the standardized protocol.

### Perfusate Sampling

Five milliliters of perfusate were sampled before commencing the HMP. Additional sampling occurred at the following perfusion time points: 5 min, 1 h, and 4 h and then at 4 h intervals in addition to an additional endpoint sample when machine perfusion ceased.

At each time point, 1 mL of fluid was also collected for point-of-care (POC) analysis (Cobas b221, Roche Diagnostics Limited) to estimate the pH, pO_2_, pCO_2_, perfusate lactate, and glucose concentrations. The remaining 4 mL of fluid was divided into two 2-mL cryovials and frozen in dry ice before storage in a –80 ^o^C freezer.

### NMR and Mass Spectrometry

Metabolite concentration was determined using 1D-^1^H NMR spectroscopy. The proportion of metabolite ^13^C incorporation in metabolites was determined using a combination of 2D-^1^H,^13^C HSQC NMR spectroscopy, and gas chromatography-coupled mass spectrometry (GC-MS).

The percentage of [U-^13^C] lactate represents the overall proportion of lactate resulting from the glycolytic metabolism of [U-^13^C] glucose within the perfusate. The experimental [U-^13^C] lactate concentration, again representing the lactate produced glycolytically from [U-^13^C] glucose within the perfusate, was calculated using the percentage of [U-^13^C] lactate and the total concentration of lactate within the perfusate. Labeled and unlabeled lactate concentrations were calculated using a combination of 2D-derived isotopomer percentages and 1D-^1^H NMR spectrum lactate concentrations. Concentrations were not adjusted according to the weight of donor organ.

Perfusate samples were prepared using our standard protocol (**Supplemental Digital Content, SDC,** http://links.lww.com/TXD/A720). 1D-^1^H NMR spectra with NOESY presat and 2D-^1^H,^13^C HSQC NMR spectra were acquired and processed using NMRPipe (version 9.2),^21^ MDDNMR (version 2.7)^22^ and MetaboLab (version 2019.12081237).^23^ The Chenomx software package (version 8.2, Chenomx Inc, Edmonton, AB) was used to quantify metabolites.

Perfusate samples were prepared for gas chromatography-coupled mass spectrometry (GC-MS) analysis (**Supplemental Digital Content, SDC,** http://links.lww.com/TXD/A720). The resulting mass spectra were analyzed using Metabolite Detector software.^24^ Finally, MetaboLab software was used to perform a combined analysis using 2D-^1^H,^13^C HSQC NMR data, and the obtained mass isotopomer distribution using the combined analysis of NMR and MS spectra approach.^25^ Full details of the preparation procedures, NMR data processing, and analysis are available in the **Supporting Information (SDC,** http://links.lww.com/TXD/A720).

### Clinical Outcomes

Delayed graft function (DGF) was defined as the need for dialysis in the first 7 d after transplantation. Recipient serum creatinine values were recorded at 1 wk, 1 mo, 6 mo, 1 y, 3 y, and 5 y postoperatively.

### Statistical Analysis

The Fisher exact test and the Mann-Whitney *U* test were used to compare groups. Unless otherwise specified, a 2-tailed Spearman’s rank correlation was used to determine whether a correlation existed for continuous data, as data sets were assumed to be nonnormally distributed. For continuous data, the median values and ranges were reported. The SDs for each fitted intensity and buildup rate were compared using an ANOVA. The study was not powered to detect differences in metabolism between DBD/DCD donor subtypes.

The statistical analyses were performed using GraphPad Prism version 8.00 for Mac OS X (GraphPad Software, La Jolla, CA) and in-house Python scripts built using SciPy (version 1.10.1). Analyses were deemed statistically significant when the *P* value was <0.05.

## RESULTS

### Donor and Recipient Characteristics

Sixteen deceased donor kidneys were perfused with [U-^13^C] glucose KPS-1. Following assessment after a period of HMP, 1 kidney was deemed unsuitable for transplantation for anatomical reasons. Another kidney was excluded because graft nephrectomy was required in the immediate postoperative period. Accordingly, 14 kidneys were included in the analysis. The median donor age was 33.2 y (range, 18.1–73.3), and the median recipient age was 37.6 y (range, 17.6–62.6), which were younger than the national and local cohorts. The donor cohort comprised 10 DBD donor kidneys (71.4%) and 4 DCD kidneys (28.6%). All donors were of White race, with equal numbers of men and women. Of the recipient cohort, 10 (71.4%) were men and 8 (57.1%) were of White race. Six patients (42.9%) had a history of hypertension and 3 (21.4%) had a history of diabetes. Table 1 and **Table S1 (SDC,** http://links.lww.com/TXD/A720) present baseline demographics in full. There were no statistical differences in demographic variables between the DBD and DCD groups.

**TABLE 1.** Demographic variables of the study population with subgroup analysis according to development of donor subtype

	All (N = 14)	DBD (N = 10)	DCD (n = 4)	*P*
	n = 14	n = 10	n = 4	
Transplant demographics				
Total CIT, h	20.4 (14.7–24.4)	19.6 (14.7–24.4)	21.5 (15.3–23.3)	0.3736
Duration of HMP, h	8.7 (5.6–13.6)	8.08 (5.58–13.6)	10.8 (7.92–11.8)	0.2398
HMP as % of total CIT	48.5 (34.3–65.5)	42.9 (34.3–65.5)	50.1 (48.4–54.7)	0.3736
Outcome				
Incidence of DGF	28.6% (n = 4)	20% (n = 2)	50% (n = 2)	0.5205
Creatinine (6 mo)	122 (59–335)	122 (59–335)	123 (86–150)	0.9436
Creatinine (1 y)	129 (75–267)	129 (75–267)	122 (102–150)	0.9436
Creatinine (3 y)	103.5 (64–220)	106 (66–220)	99 (64–147)	0.5241
Creatinine (5 y)	115 (64–256)	115 (64–256)	104.5 (70–129)	0.5712

CIT, cold ischemia time; DBD, donation after brain death; DCD, donation after circulatory death; DGF, delayed graft function; HMP, hypothermic machine perfusion.

The median cold ischemia time was 20.4 h (14.7–24.4 h), and the median duration of HMP was 8.7 h (range, 5.6–13.6 h). All kidneys underwent HMP for at least 4 h, enabling consistent time-point sampling at both 1  and 4 h, in addition to the endpoint of HMP.

### Perfusion Parameters and POC Data

POC values and renal resistance during the HMP are summarized in Table 2. As anticipated, renal resistance decreased rapidly after the start of perfusion and stabilized after 1 h (mean 0.21 mm Hg/mL/min). A decrease in fluid glucose concentration was noted, from 10 mmol/L in the fluid at the commencement of HMP to 9.1 mmol (8.6–10 mmol) and 8.6 mmol (8.0–9.4 mmol) after 1 and 4 h, respectively. In addition, there was a decrease in the oxygen and pH concentrations, with a reciprocal increase in the lactate concentration during HMP.

**TABLE 2.** Changes in POC lactate, glucose, pH, and renal resistance over time

	1 h	4 h	*P*	T end
	Mean ± SD	Mean ± SD	(1 vs 4 h)	Mean ± SD
POC lactate	1.4 ± 0.35	2.05 ± 0.52	0.003	2.4 ± 0.77
POC glucose	9.15 ± 0.41	8.6 ± 0.40	0.007	8.44 ± 0.41
POC pH	7.17 ± 0.049	7.12 ± 0.052	0.016	7.10 ± 0.061
pO_2_	12.79 ± 2.63	11.81 ± 0.81	0.011	11.38 ± 0.90
Renal resistance, mm Hg/mL/min	0.21 ± 0.49	0.21 ± 0.47	>0.99	0.22 ± 0.32

All metabolite concentrations units mmol/L unless otherwise stated.

POC, point of care.

### Untargeted Metabolomics

**FIGURE 1.**
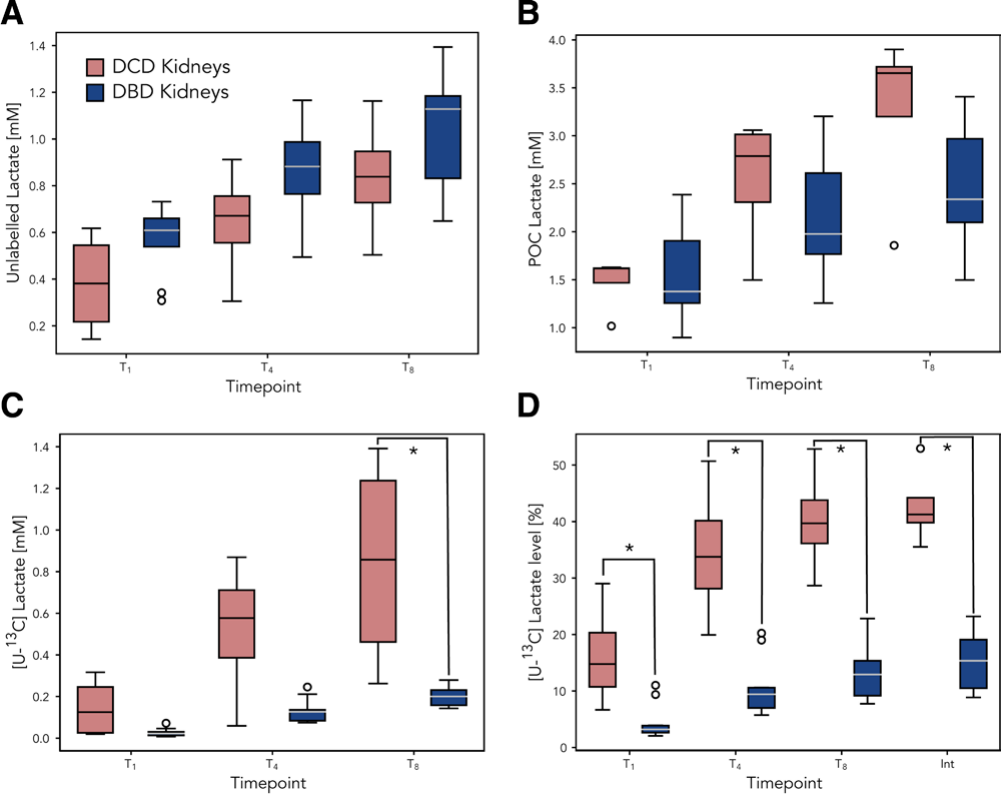
Box and Whisker plot demonstrating differences in [U-^12^C] lactate concentrations (A), point-of-care lactate concentrations (B), [U-^13^C] lactate concentrations (C), and [U-^13^C] lactate percentages (D) between DBD and DCD kidneys. DCD kidneys are shown in red and DBD kidneys are shown in blue. Asterisks indicate statistically significant differences between DBD and DCD kidneys (*P* < 0.05). DBD, donation after brain death; DCD, donation after circulatory death; POC, point of care.

1D-^1^H NMR spectroscopic analysis of the HMP perfusate identified a panel of 22 consistently present metabolites. These results are similar to those documented in our previous studies (**Table S2 SDC,** http://links.lww.com/TXD/A720). During HMP, the alanine, lactate (Figure 1A and B), and glutamate concentrations increased between the 1 and 4 h time points, with a reciprocal decrease in glutathione level.

### Tracer-based Metabolism

**FIGURE 2.**
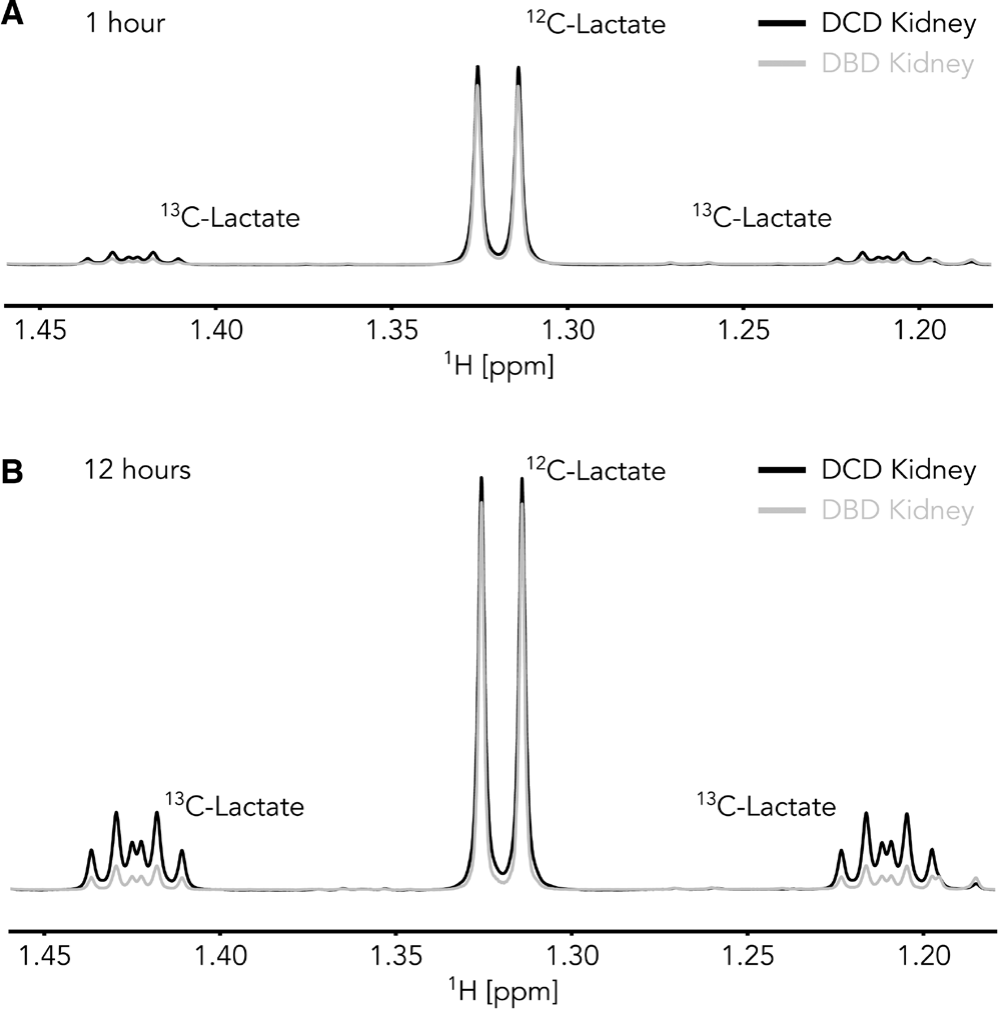
Lactate formation during hypothermic machine perfusion at 1 h (A) and 12 h (B) of perfusion. The central doublet peak reflects the amount of [U-^12^C] lactate (ie, unlabeled lactate), whereas the more complex satellite signals on either side of the central doublet are caused by [U-^13^C] lactate. DCD kidney data are shown in black, whereas DBD kidney data are plotted in gray. DBD, donation after brain death; DCD, donation after circulatory death.

**FIGURE 3.**
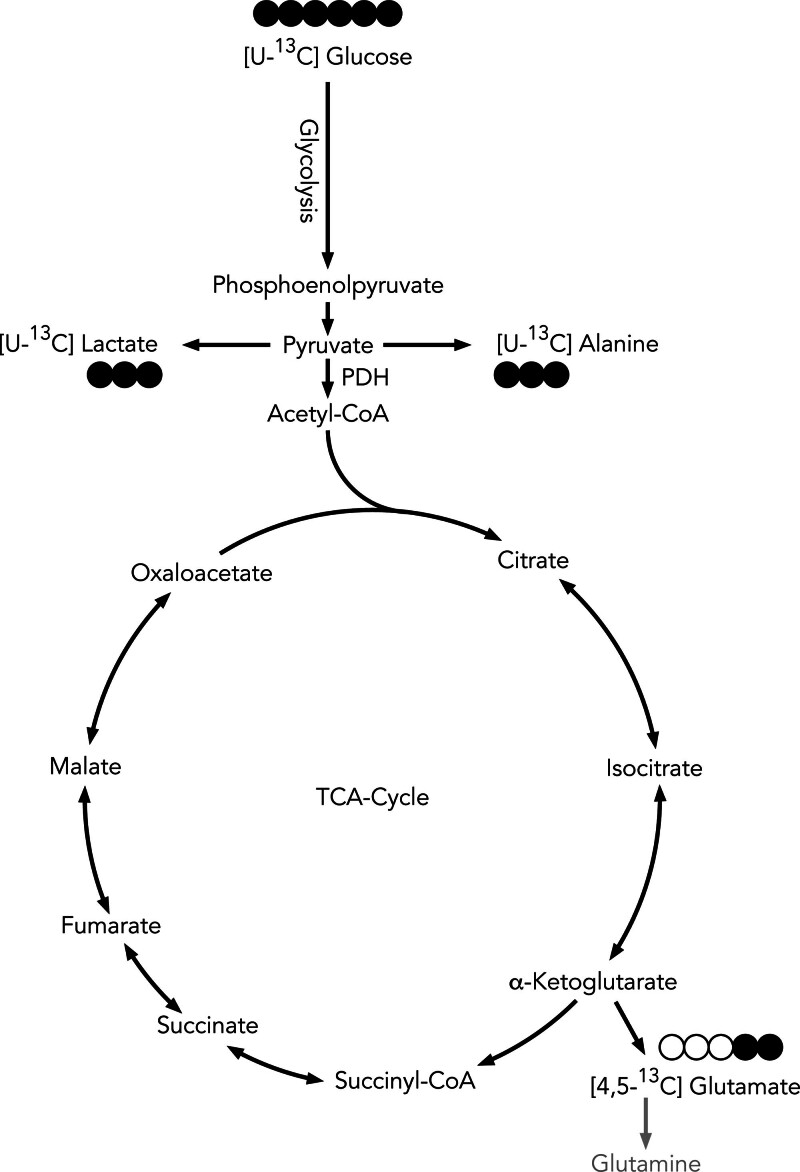
Glucose metabolism. [U-^13^C] glucose is metabolized via glycolysis to form pyruvate. Pyruvate can then be reduced to form lactate, transaminated to form the amino acid alanine or enter the TCA cycle. A popular read-out for TCA cycle activity is the amino acid glutamate. If synthesized from [U-^13^C] glucose, this would form [4,5-^13^C] glutamate. 4,5-^13^C, glutamate labeled in carbons 4 and 5; Acetyl-CoA, acetyl coenzyme A; PDH, Pyruvate Dehydrogenase; TCA, tricarboxylic acid .

Using 2D-^13^C,^1^H HSQC NMR spectroscopy and GC-MS analysis, ^13^C incorporation was quantified for lactate (Figure 1C and D) and alanine. This technique provided evidence of ^13^C metabolite incorporation over and above the naturally occurring baseline level (1.07%), even after 1 h of perfusion (Figure 2). This highlights that within a relatively short period, the cellular uptake of circulating glucose occurs, leading to metabolite incorporation with subsequent transport/delivery back into the circulating fluid (Figure 3). For example, the increase in lactate concentrations appeared to be associated with an increase in ^13^C-labeled glucose utilization (Figure 1C and D).

Table 3 and Figure 1B show the perfusion fluid lactate measurements according to the donor type. During HMP, it was found that not only did the overall (^13^C and ^12^C) concentration of lactate (Figure 1A) increase in the perfusion fluid but also the proportion of the ^13^C isotope (Figure 1C and D; **Figure S1, SDC,** http://links.lww.com/TXD/A720). Thus, there was a dramatic increase in the calculated concentration of ^13^C-labeled lactate during perfusion, reflecting *de novo* glycolytic pathway activity in all kidneys reviewed.

**TABLE 3.** Lactate and alanine labeling and levels DBD vs DCD

Time point, h	Percentage of U^13^C lactate, %			Experimental [U-^13^C] lactate concentrations, mM			POC lactate Concentrations, mM		
	DBD (N = 10)	DCD (N = 4)	*P*	DBD (N = 10)	DCD (N = 4)	*P*	DBD (N = 10)	DCD (N = 4)	*P*
1	3.135 ± 2.969	14.714 ± 8.277	0.008	0.019 ± 0.014	0.093 ± 0.091	0.260	1.300 ± 0.396	1.500 ± 0.218	1.000
4	9.362 ± 4.818	33.722 ± 11.108	0.004	0.094 ± 0.039	0.417 ± 0.213	0.188	1.800 ± 0.491	2.475 ± 0.521	0.395
8	12.867 ± 4.714	39.677 ± 8.608	0.002	0.147 ± 0.032	0.618 ± 0.332	0.004	2.100 ± 0.468	3.195 ± 0.682	0.135
∞ (Intensity)	15.302 ± 4.808	41.249 ± 6.342	0.002						
	Percentage of U^13^C alanine, %			Experimental [U-^13^C] alanine concentrations, mM					
1	0.701 ± 2.183	5.729 ± 6.942	0.199	0.0002 ± 0.0007	0.0027 ± 0.0033	0.199			
4	5.281 ± 6.802	18.067 ± 8.763	0.028	0.0047 ± 0.0051	0.0116 ± 0.0076	0.076			
8	8.369 ± 7.717	22.480 ± 9.855	0.199	0.0081 ± 0.0052	0.0176 ± 0.0122	0.076			

DBD, donation after brain death; DCD, donation after circulatory death; POC, point of care.

Similarly, there was an increase in the percentage of labeled alanine during perfusion for both DBD and DCD kidneys, with an accompanying increase in absolute levels and total calculated concentration of ^13^C-labeled alanine, again reflecting glycolytic pathway activity.

However, a striking difference between *de novo* lactate formation in DBD and DCD kidneys was observed, with a significantly higher proportion of [U-^13^C] lactate in DCD kidneys from the start of machine perfusion (T_1_, T_4_, and T_8_; Figure 1D). After 8 h of perfusion, there was more than a 4-fold difference in the calculated [U-^13^C] lactate concentration between the DCD and DBD groups (0.62 versus 0.15 mM, *P* = 0.004; Figure 1C) indicative of significantly higher glycolytic pathway activity in DCD kidneys.

Despite the increased concentration of glutamate and the decreasing concentration of oxygen levels in the perfusion fluid (**Figure S2, SDC,** http://links.lww.com/TXD/A720), no ^13^C-labeled glutamate and hence no convincing evidence of *de novo* aerobic metabolism was detected in this study. Furthermore, there was no association between the concentration of labeled metabolites and the duration of HMP, SCS, or total cold ischemia time.

### Clinical Outcomes

Recipients of the 14 kidneys included in this study have, to date, exhibited 100% patient and graft survival rates. The median serum creatinine values at 1, 3, and 5 y were 129, 104, and 125 µmol/L, respectively, with no statistically significant difference between creatinine levels in the DBD and DCD kidneys at 6 mo, 1 y, 3 y, or 5 y (Table 1).

There was no association between the proportion of [U-^13^C] lactate labeling, [U-^13^C] lactate concentrations, experimental absolute lactate concentrations (from NMR data), or POC lactate concentrations and the incidence of DGF (**Table S3, SDC,** http://links.lww.com/TXD/A720). Patients with DGF had higher serum creatinine values than those with immediate graft function (**Table S4, SDC,** http://links.lww.com/TXD/A720). In short, none of the measured lactate parameters correlated with early graft function.

There was also no correlation between 8-h [U-^13^C] lactate concentration and 6-mo, 1-y, 3-y, or 5-y creatinine levels (**Table S5, SDC,** http://links.lww.com/TXD/A720).

## DISCUSSION

In this first-in-human study, we have unequivocally demonstrated that human deceased donor kidneys considered suitable for transplantation exhibited low levels of metabolic activity during hypothermic perfusion. Moreover, *de novo* metabolites indicating glycolytic pathway activity as reflected in [U-^13^C] lactate production occurring during HMP differs between the DBD and DCD kidneys. This likely reflects the warm ischemic insult to DCD kidneys during the retrieval process.

Hence, using tracer-based metabolism, we have demonstrated that the ongoing consequences of this initial ischemic insult persist during HMP. The presence of unlabeled lactate, indicating anaerobic metabolism, in the perfusate immediately after commencing HMP likely represents the washout of excess intracellular lactate that accumulates during pre-HMP SCS, as the global (total) lactate concentrations in all kidneys were initially similar. Ischemic conditions are known to cause a build up of intracellular lactate, resulting in acidosis and cellular damage. These metabolic consequences are central to the ischemia/reperfusion injury (IRI) phenomenon.

Subsequent *de novo* lactate production derived from perfusate [U-^13^C] glucose was evident in all DBD and DCD kidneys soon after commencing HMP, indicating ATP production via glycolytic pathway activity. Such results, indicating ongoing metabolism after the commencement of HMP, reinforce the need to provide a metabolic substrate during hypothermic conditions. By extension, interventions to support metabolic pathways during HMP could be expected to regenerate ATP stores via alternative pathways, optimizing organ pre-transplantation.

### Differences in DBD Versus DCD Kidneys

After an initial washout phase, modeling of the [U-^13^C] lactate data revealed a clear difference in the ongoing lactate production between DBD and DCD kidneys during the HMP period; despite small numbers, DCD kidneys seemed to produce much more lactate derived from the glucose in the perfusion fluid compared with DBD kidneys.

In DCD kidneys, there was a sustained increase in the conversion of [U-^13^C] glucose into the glycolytic end-product lactate ([U-^13^C] lactate). Such a difference between DBD and DCD kidneys was visible as early as 1 h after commencing HMP as evidenced by significantly higher percentages of ^13^C enrichment in lactate. Later, this was reflected in the higher plateau concentration of *de novo* [U-^13^C] lactate in the DCD organs. Such changes were notable and consistent despite a small overall sample size with a low number of DCD kidneys (n = 4). The observed difference occurred independent of oxygen availability.

### Explanation for Differences in DBD and DCD Kidneys

The explanation for such differences likely stems from donor organ physiology during the retrieval process. After cessation of blood flow during a warm ischemic period at the time of donor asystole, intracellular glycogen stores are depleted in DCD kidneys. Hence DCD kidneys likely display a relative thirst for glucose after the initiation of HMP. This may be achieved via an increase in their capacity to take up extracellular glucose by upregulating monocarboxylate transporters responsible for transporting lactate into the perfusate on a cellular level.

An alternative explanation is that higher [U-^13^C] lactate concentrations in DCD kidneys reflect a switch to energy production via glycolysis due to warm ischemia, and conventional HMP for short periods does not provide an opportunity to reverse such a metabolic switch without additional oxygenation.

### Immunological Explanation

An alternative explanation for the increase in [U-^13^C] lactate buildup is possibly due to the elevated glycolytic activity of other cells present in the donor kidney. This may include resident innate immune cells because it is now well established that immune cells substantially alter their metabolic pathway activity on relevant stimulation and particularly increase the uptake of glucose and the conversion of glucose-derived pyruvate to lactate. This metabolic change supports immune functions such as inflammatory mediator production by increasing the abundance of glycolytic intermediates available for protein, nucleic acid, and lipid synthesis.

Such processes act may act as a precursor to IRI as may have implications for the recipient with regards to immune responses in the transplant recipient. Due to the number of immune cells that are resident in donor organs, they are unlikely to be the main contributor to *de novo* lactate production. However, further studies are required to fully understand the extent to which damaged organs can provoke an immune response in differing preservation conditions.

### Functional Outcomes

The lack of correlation between *de novo* lactate production and functional outcomes suggests that the metabolic signature of deceased donor kidneys during HMP does not have long-erm implications. This is in keeping with the equivalence of functional outcomes of DBD and DCD kidneys in transplantation despite contrasting *de novo* metabolic activity observed during HMP in our small cohort of kidneys. Alternatively, this negative finding may be due to sample size or the resilience of a relatively younger cohort of donors and recipients to IRI compared with the general donor and recipient pool in transplantation.

### Active Oxygenation During Organ Retrieval and Organ Preservation

Given the evidence of glycolytic pathway activity, it could reasonably be inferred that perfusate oxygenation would have been beneficial in gaining a higher ATP yield for each molecule of glucose and avoiding the implications of lactate buildup due to anaerobic metabolism. This study was specifically conducted using standard HMP to provide information on baseline physiology without the addition of oxygenation. Our previous study using [U-^13^C] glucose in a similar methodology demonstrated the benefits of additional oxygenation during HMP provided by a membrane oxygenator in a paired ex vivo porcine model, showing an increase in *de novo* TCA cycle activity, better preservation of mitochondrial architecture, and regeneration of ATP levels when the perfusate is oxygenated compared with simple aeration.^20^ More straightforward means of oxygenation during HMP include bubble and surface oxygenation, which achieve partial pressures of oxygen similar to those in membrane-oxygenated kidneys.^26^

Active oxygen delivery during HMP has been studied in the clinical setting during the entire duration of HMP in DCD kidneys^27^ (versus conventional HMP) and for a 2-h period toward the end of a period of SCS in expanded criteria donor (ECD) kidneys.^28^ Jochmans et al^27^ found that oxygenation during HMP resulted in significantly lower graft failure and less severe complications. Even so, neither study design showed a marked clinical benefit in reducing the incidence of DGF.

### Timing of Initiation of HMP

In this study, the kidneys underwent HMP for a median of 8.7 h. This relatively short duration contrasts with 18 h of HMP in our previous metabolomic and metabolic tracing studies.^15,19,29^ In contrast to the products of glycolysis, which can appear within minutes, TCA cycle intermediates such as glutamate do not appear for several hours.^20^ This could be one reason why [4,5-^13^C] glutamate was not detected in this analysis, evidencing TCA cycle activity, as observed in previous porcine studies where oxygenation was provided during HMP.^20,30,31^

In the current study population, the kidneys underwent HMP for less than half of the total CIT, with a median duration of 8.4 h of SCS before initiating HMP. From a metabolism perspective, our data support the use of HMP earlier in the preservation period for DCD kidneys compared with SCS.

### Limitations

The main limitation of our study was the sample size. The small number of organs perfused was due to constraints on performing such studies using human tissue. The conclusions drawn were limited by the inevitable multiple variables in clinical transplantation. Yet even with the limitation of sample size, the differences between DCD and DBD kidneys were still apparent and consistent.

Finally, it is clear that NMR spectroscopy can provide important information regarding metabolism in ex vivo organs before transplantation. However, due to the length of sample processing times, it is unlikely to be clinically useful in real time for organ assessment/viability during perfusion, although previous studies have used 1D NMR spectroscopy for this purpose.^16^

## CONCLUSIONS

This study provides unequivocal evidence of *de novo* anaerobic metabolism occurring even when organs are stored under hypothermic conditions and in the absence of perfusate oxygenation. As such, it highlights the importance of supporting organs metabolically during this period of subphysiological temperature through the delivery of readily available glucose substrate. Failure to do so may risk intracellular lactate buildup and subsequent tissue damage.

The study also highlights the metabolic differences between DBD and DCD kidneys. By the time of HMP initiation, DCD kidneys are likely starved of oxygen after a period of warm ischemia. Intracellular glucose levels are likely to be depleted because of reliance on glycolysis during organ retrieval and the hypoxic conditions of static storage. Using tracer-based metabolism, we have demonstrated that the ongoing consequences of this initial ischemic insult persist during HMP and would suggest that means to reverse this situation are included following the commencement of HMP.

## ACKNOWLEDGMENTS

The authors thank Renal Transplant Surgeons and operating theater staff at the Queen Elizabeth Hospital Birmingham in addition to William Ries, Charlotte Turnbull, and Ohema Powell for their assistance with experimental research.

## Supplementary Material


