# Supplementary material for: Metabolic Characterization of Deceased Donor Kidneys Undergoing Hypothermic Machine Perfusion Before Transplantation Using 13C-enriched Glucose
Source: Transplant Direct. 2024 Dec 10;11(1):e1736. doi: 10.1097/TXD.0000000000001736 (PMC11634324; doi:10.1097/TXD.0000000000001736)
Supplement: Supplementary file 1 [file txd-11-e1736-s001.pdf]

Supplemental Digital Content

Metabolic characterisation of deceased donor kidneys undergoing Hypothermic Machine Perfusion prior to transplantation using ^{13}C enriched glucose

Kamlesh Patel, Jay Nath, Thomas Smith, Tom Darius, Alpesh Thakker, Sarah Dimeloe, Nicholas Inston, Andrew Ready, Christian Ludwig

Sample preparation and metabolite determination using NMR Spectroscopy and Mass Spectrometry

Perfusate samples were thawed before vortexing for 30 seconds. 430µL of perfusate were then removed from each 2ml cryovial in preparation for NMR sample preparation. Processing involved mixing with 200µL 400 mM phosphate buffer, containing 2 mM Tri-methyl silyl d5-propionate, sodium salt (TMSP) and 40% D_2O with further mixing via vortexing and centrifugation before placing 600µL in a 5mm NMR tube. Both 1D-^{1}H NMR and 2D-^{1}H,^{13}C heteronuclear single quantum coherence (HSQC) NMR spectra were acquired using a 600-MHz Bruker Avance III NMR spectrometer, equipped with a TCI cryogenic probe, equipped with a z-axis pulsed field gradient. From each sample cryovial, 43µL was also removed for analysis by mass spectrometry.

1D-^{1}H NMR free induction decays (FIDs) were apodised using 0.3 Hz exponential line-broadening and zero-filled to 131072 real data points before Fourier transform to obtain the actual NMR spectra. The resulting output NMR spectra were then manually phase corrected, automatically referenced to the added internal standard TMSP and baseline corrected using spline baseline correction[1]. The spectra were then exported to Bruker format for metabolite concentrations to be quantified using Chenomx 8.2 (Chenomx INC, Edmonton, AB, Canada). Final metabolite concentrations accounted for dilution with NMR buffer during sample preparation. 2D-^{1}H,^{13}C HSQC NMR spectra were zero-filled to 1024 real data points for the ^{1}H dimension. The spectra were acquired using non-uniform sampling (NUS) and therefore 2D-NMR spectra were reconstructed using the IRLS algorithm with NMRPipe[2] (ver. 9.2) and MDDNMR[3] (version 2.7). The resulting 2D-NMR spectra were referenced to the methyl group resonance of lactate.

For GC-MS (Gas Chromatography coupled Mass Spectrometry) analysis, the dried polar extract was dissolved in 2% methoxyamine HCl in pyridine (Sigma-Aldrich, Dorset, UK) followed by incubation at 60°C and subsequently 60 µL Ntertbutyldimethylsilyl-N-methyltrifluoroacetamide with 1% (w/v) tertbutyldimethyl-chlorosilane (Sigma-Aldrich, Dorset, UK) derivatization reagent was added. GC-MS was performed using an Agilent 7890B Series GC/MSD gas chromatograph with a polydimethylsiloxane GC column coupled, with a mass spectrometer (Agilent Technologies UK Limited, Stockport, UK). For the determination of the mass isotopomer distributions, spectra were corrected for natural isotope abundance. Data processing from raw spectra to mass isotopomer distribution correction and determination was performed using Metabolite Detector software[4].

The Matlab based MetaboLab software (version 2019.12081237)[1] was used to combine data from 2D-^{1}H,^{13}C HSQC NMR and Gas Chromatography Mass Spectrometry (GC-MS) using the CANMS approach[5] (10) to give accurate isotopomer distributions for key metabolites. The total concentrations of alanine and lactate were determined by adjusting ^{1}H NMR concentrations, taking into account isotopomer distributions as determined by 2D-^{1}H,^{13}C HSQC NMR and GC-MS analysis.

Supplemental Figures

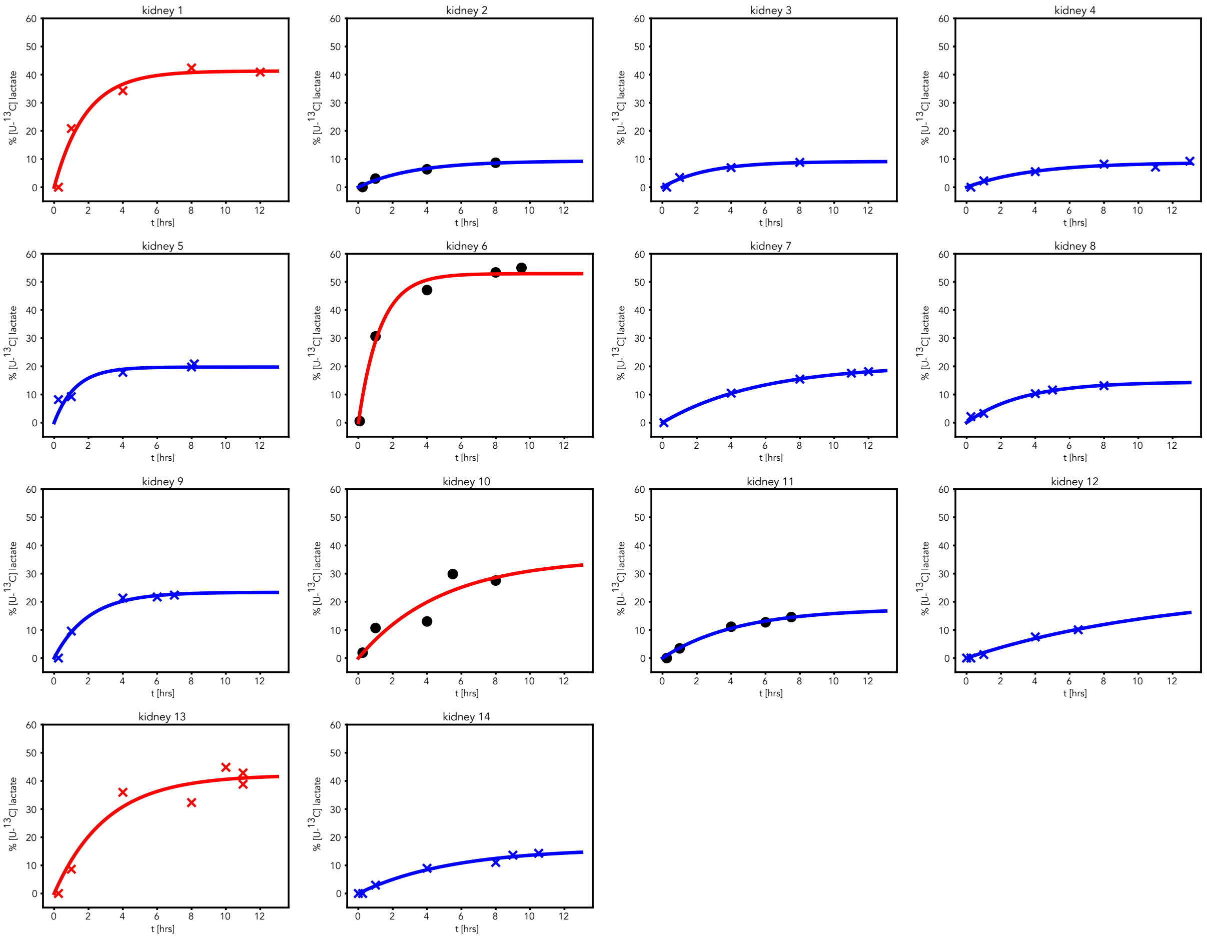

Figure S1. [U-^{13}C] Lactate build-up in all fourteen kidneys. [U-^{13}C] lactate percentages were calculated through combined analysis of 2D-^{1}H,^{13}C HSQC NMR Spectroscopic and GC-MS data[5]. The ^{13}C-NMR percentages were then fitted to a mono-exponential build-up curve using in-house Python scripts. DCD kidneys are plotted in red, and DBD kidneys are plotted in blue. Experimental percentage values of DGF kidneys are plotted using black dots, those of IGF kidneys with blue or red crosses.

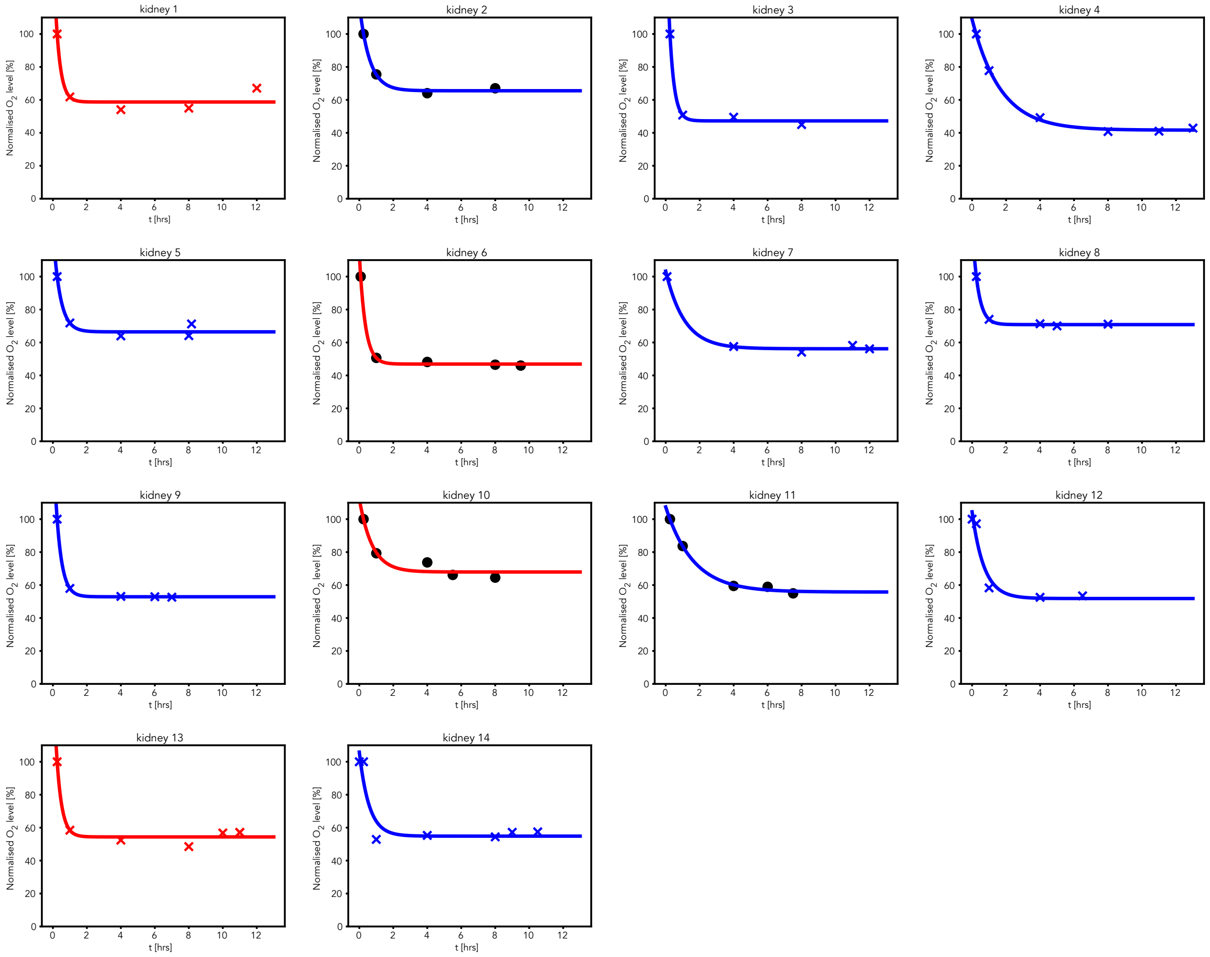

Figure S2. POC O_2 levels were fitted to a mono-exponential decay using in-house Python scripts. DCD kidneys are plotted in red, and DBD kidneys are plotted in blue. Experimental O_2 levels of DGF kidneys are plotted using black dots, those of IGF kidneys with blue or red crosses.

References

1. Ludwig C, Günther UL. MetaboLab - advanced NMR data processing and analysis for metabolomics. *BMC Bioinformatics*. 2011;12(1):366. doi:10.1186/1471-2105-12-366

2. Delaglio F, Grzesiek S, Vuister GeertenW, Zhu G, Pfeifer J, Bax A. NMRPipe: A multidimensional spectral processing system based on UNIX pipes. *J Biomol NMR*. 1995;6(3). doi:10.1007/BF00197809

3. Kazimierczuk K, Orekhov VYu. Accelerated NMR Spectroscopy by Using Compressed Sensing. *Angew Chem Int Ed*. 2011;50(24):5556-5559. doi:10.1002/anie.201100370

4. Hiller K, Hangebrauk J, Jäger C, Spura J, Schreiber K, Schomburg D. MetaboliteDetector: Comprehensive Analysis Tool for Targeted and Nontargeted GC/MS Based Metabolome Analysis. *Anal Chem*. 2009;81(9):3429-3439. doi:10.1021/ac802689c

5. Chong M, Jayaraman A, Marin S, et al. Combined Analysis of NMR and MS Spectra (CANMS). *Angew Chem Int Ed*. 2017;56(15):4140-4144. doi:10.1002/anie.201611634

Table S1. Full demographic variables of study population with subgroup analysis according to development of donor sub-type

	All n=14	DBD n=10	DCD n=4	p-value
**Donor demographics**				
Male	50.0% (n=7)	40% (n=4)	75% (n=3)	0.5594
Age (yrs)	33.2 (18.1-73.3)	33.2 (18.1 - 65.5)	36.6 (21.5 - 73.3)	0.8392
DCD donor	28.6% (n=4)	n/a	n/a	n/a
Caucasian	100% (n=14)	100% (n=10)	100% (n=4)	>0.9999
Traumatic brain injury	21.4% (n=3)	30.0% (n=3)	0% (n=0)	0.5055
History of hypertension	21.4% (n=3)	20% (n=2)	25% (n=1)	>0.9999
History of diabetes	0% (n=0)	0% (n=0)	0% (n=0)	>0.9999
BMI (kg/m^2)	23.7 (17.1-43.0)	22.66 (17.1 - 28.1)	28.0 (23.3 - 43)	0.1419
**Recipient demographics**				
Male	71.4% (n=10)	70% (n=7)	75% (n=3)	>0.9999
Age (yrs)	37.6 (17.6-62.6)	33.5 (17.6 - 56.4)	46.4 (31.4 - 62.6)	0.1419
BMI (kg/m^2)	29.7 (18.2-33.8)	29.5 (18.2 - 33.8)	30.4 (21.9 - 33.8)	0.4535
Caucasian	57.1% (n=8)	60.0% (n=6)	50% (n=2)	>0.9999
History of hypertension	42.9% (n=6)	30.0% (n=3)	75% (n=3)	0.2448
History of diabetes	21.4 (n=3)	20.0% (n=2)	25.0% (n=1)	>0.9999
CMV positive	42.9% (n=6)	30.0% (n=3)	75% (n=3)	0.2448
**Transplant demographics**				
Total CIT (hrs)	20.4 (14.7-24.4)	19.6 (14.7 - 24.4)	21.5 (15.3 - 23.3)	0.3736
Duration of HMP (hrs)	8.7 (5.6-13.6)	8.08 (5.58 - 13.6)	10.8 (7.92 - 11.8)	0.2398
HMP as % of total CIT	48.5 (34.3-65.5)	42.9 (34.3 - 65.5)	50.1 (48.4 - 54.7)	0.3736
**Outcome**				
Incidence of DGF	28.6% (n=4)	20% (n=2)	50% (n=2)	0.5205

Table S2. Differences in metabolite concentration between DBD and DCD donor kidneys. All metabolite concentrations are displayed as median ± standard deviation in [mM]

	1 hour			4 hours			T End		
	**DBD** (n=10) [mM]	**DCD** (n=4) [mM]	p-value	**DBD** (n=10) [mM]	**DCD** (n=4) [mM]	p-value	**DBD** (n=10) [mM]	**DCD** (n=4) [mM]	p-value
Methylxanthine	0.027 ± 0.003	0.026 ± 0.002	0.357	0.028 ± 0.003	0.025 ± 0.002	0.203	0.027 ± 0.004	0.027 ± 0.002	0.839
Acetate	0.210 ± 0.125	0.185 ± 0.128	0.945	0.194 ± 0.115	0.172 ± 0.113	0.945	0.172 ± 0.093	0.170 ± 0.107	0.944
Adenine	5.770 ± 0.399	5.869 ± 0.344	0.635	5.495 ± 0.515	5.727 ± 0.229	0.374	5.846 ± 0.407	5.320 ± 0.276	0.240
Alanine	0.070 ± 0.018	0.057 ± 0.018	0.374	0.129 ± 0.048	0.081 ± 0.018	**0.024***	0.153 ± 0.053	0.113 ± 0.031	0.106
Ethanolamine	7.350 ± 0.721	6.540 ± 0.296	**0.024***	7.122 ± 1.278	7.669 ± 1.061	0.539	7.532 ± 1.296	7.776 ± 0.552	0.539
Formate	0.182 ± 0.102	0.156 ± 0.108	0.839	0.199 ± 0.114	0.177 ± 0.112	0.945	0.207 ± 0.106	0.150 ± 0.083	0.733
Fumarate	0.002 ± 0.001	0.004 ± 0.002	0.288	0.003 ± 0.002	0.005 ± 0.002	0.179	0.005 ± 0.002	0.008 ± 0.003	0.089
Gluconate	76.23 ± 6.50	76.65 ± 1.07	0.839	76.98 ± 6.23	78.94 ± 1.66	0.839	79.70 ± 5.72	78.74 ± 2.67	>0.999
Glutamate	0.303 ± 0.094	0.350 ± 0.140	0.539	0.943 ± 0.206	1.113 ± 0.372	0.733	1.518 ± 0.425	2.033 ± 0.732	0.454
Glutathione	1.344 ± 0.185	1.297 ± 0.062	0.454	0.835 ± 0.244	0.748 ± 0.139	0.671	0.576 ± 0.187	0.116 ± 0.361	0.304
Glycine	0.430 ± 0.334	0.783 ± 0.296	0.945	1.543 ± 0.679	2.149 ± 0.750	0.539	2.791 ± 0.987	3.312 ± 1.040	0.374
Hippurate	0.004 ± 0.402	0.000 ± 0.001	0.051	0.005 ± 0.005	0.001 ± 0.001	0.128	0.009 ± 0.005	0.000 ± 0.000	0.036*
Hypoxanthine	0.104 ± 0.058	0.101 ± 0.068	0.721	0.175 ± 0.103	0.200 ± 0.077	>0.999	0.184 ± 0.114	0.190 ± 0.063	0.945
Inosine	0.026 ± 0.024	0.014 ± 0.016	0.539	0.034 ± 0.035	0.010 ± 0.025	0.203	0.024 ± 0.022	0.005 ± 0.016	0.077
Isopropanol	0.014 ± 0.005	0.014 ± 0.001	0.944	0.015 ± 0.002	0.015 ± 0.001	0.478	0.016 ± 0.003	0.016 ± 0.002	0.777
Lactate	0.937 ± 0.217	0.675 ± 0.373	0.454	1.528 ± 0.294	1.459 ± 0.540	0.839	1.907 ± 0.374	1.759 ± 1.163	>0.999
Leucine	0.010 ± 0.004	0.005 ± 0.005	0.188	0.017 ± 0.008	0.011 ± 0.006	0.157	0.019 ± 0.012	0.011 ± 0.006	0.374
Oxypurinol	0.293 ± 0.033	0.283 ± 0.027	0.539	0.322 ± 0.029	0.308 ± 0.016	0.396	0.312 ± 0.048	0.310 ± 0.028	0.635
Ribose	1.594 ± 0.183	1.594 ± 0.032	0.210	1.594 ± 0.167	1.594 ± 0.018	>0.999	1.594 ± 0.232	1.723 ± 0.148	0.556
Succinate	0.018 ± 0.005	0.018 ± 0.004	0.733	0.018 ± 0.006	0.023 ± 0.008	0.188	0.025 ± 0.008	0.025 ± 0.017	0.839
Tyrosine	0.006 ± 0.003	0.015 ± 0.122	0.055	0.011 ± 0.166	0.009 ± 0.003	0.288	0.012 ± 0.124	0.012 ± 0.005	>0.999
Valine	0.008 ± 0.004	0.007 ± 0.003	0.395	0.013 ± 0.007	0.012 ± 0.004	0.539	0.015 ± 0.009	0.016 ± 0.006	0.436

Table S3. Fitted ^{13}C lactate percentages, experimental [U-^{13}C] lactate concentrations, experimental absolute lactate concentrations (from NMR data), and POC lactate concentrations for DFG and non-DFG kidneys. P-values <0.05 are deemed significant, highlighted in bold, and marked with an asterisk (*).

Fitted ^{13}C lactate levels [%]			
Timepoint [hrs]	DGF (n=4)	Non-DGF (n=10)	p-values
1	5.147 ± 10.806	3.596 ± 5.092	0.733
4	15.231 ± 17.271	10.340 ± 10.287	0.635
8	21.596 ± 17.038	14.405 ± 11.266	0.635
∞ (Intensity)	26.232 ± 16.918	17.930 ± 11.111	0.539
Experimental [U-^{13}C] lactate concentrations [mM]			
Timepoint [hrs]	DGF (n=4)	Non-DGF (n=10)	p-values
1	0.018 ± 0.093	0.024 ± 0.045	0.710
4	0.101 ± 0.343	0.100 ± 0.134	0.839
8	0.168 ± 0.371	0.164 ± 0.216	>0.999
Experimental lactate concentrations [mM]			
Timepoint [hrs]	DGF (n=4)	Non-DGF (n=10)	p-values
1	0.414 ± 0.182	0.613 ± 0.153	0.106
4	0.940 ± 0.196	0.898 ± 0.204	0.733
8	0.864 ± 0.217	1.150 ± 0.240	0.304
POC lactate concentrations [mM]			
Timepoint [hrs]	DGF (n=4)	Non-DGF (n=10)	p-values
1	1.350 ± 0.212	1.500 ± 0.388	0.484
4	1.950 ± 0.495	2.100 ± 0.530	0.944
8	2.150 ± 0.579	2.400 ± 0.622	0.722

Table S4. Median creatinine values ± standard deviations for DGF and non-DGF kidneys, six months, and 1, 3 and 5 years post-surgery.

Timepoint	median ± stddev (DGF)	median ± stddev (non-DGF)	p-values
6 months	146.000 ± 93.799	112.500 ± 25.472	0.288
1 year	147.500 ± 59.199	119.500 ± 21.412	0.065
3 years	126.500 ± 56.764	94.000 ± 24.744	0.524
5 years	136.000 ± 67.759	103.500 ± 23.771	0.288

Table S5. Pearson correlation coefficients and coefficient of determination values for creatinine values and 8 hour [U-^{13}C] lactate concentrations.

Timepoint	Pearson correlation coefficient	Coefficient of determination
6 months	-0.293	0.086
1 year	-0.216	0.047
3 years	-0.383	0.147
5 years	-0.344	0.119
